# Formulation and Evaluation of Quality Parameters of Effervescent Granules from the Potent Antioxidant between Two Variants of the Adaptogenic Herb *Ocimum tenuiflorum* L.

**DOI:** 10.1155/2023/2050846

**Published:** 2023-04-25

**Authors:** Kalpana Parajuli-Baral

**Affiliations:** School of Health and Allied Sciences, Faculty of Health Sciences, Pokhara University, Pokhara 33700, Kaski, Nepal

## Abstract

*Ocimum tenuiflorum* L. is found throughout semitropical and tropical parts of Southeast Asia. In Nepal, *O. tenuiflorum* L. is popular with two variants: Krishna Tulsi consisting of purple-colored leaves and Sri Tulsi consisting of green-colored leaves. *O. tenuiflorum* L. is considered the queen of herbs and is a traditionally and clinically proven medicinal herb for its application and efficacy. However, no commercial pharmaceutical preparations of *O. tenuiflorum* L. are available using effervescent vehicles. Therefore, the present study aimed to compare the antioxidant activity of leaves from the two varieties of *O. tenuiflorum* L. and formulate and evaluate the quality parameters of effervescent granules of the potent extract. The antioxidant activity of *O. tenuiflorum* L. ethanolic extracts was evaluated by DPPH radical scavenging assay at 1, 10, and 100 *µ*g/mL concentrations, where ascorbic acid was used as the positive control. The antioxidant activity of purple-leafed *O. tenuiflorum* L. was found to be higher than that of green-leafed *O. tenuiflorum* L. Therefore, effervescent granules of the ethanolic extract of purple-leafed* O. tenuiflorum* L. were formulated using the pharmaceutical excipients tartaric acid, citric acid, and sodium bicarbonate and the quality parameters of the granules were evaluated. The formulated granules met the quality parameters assessed from the angle of repose, bulk density, tapped density, Carr's Index, Hausner's ratio, effervescent cessation time, and stability studies. Thus, the formulated effervescent granules of *O. tenuiflorum* L. can be used for therapeutic purposes or as a functional food.

## 1. Introduction

From past to present, the use of plants has always maintained its importance. The most striking part of these usage areas is pharmacology [1–3]. Recently, lifestyle-related chronic diseases have become the prevailing causes of global morbidity and mortality, many of which could be managed through Ayurveda with its primary focus on the regular consumption of adaptogenic herbs and healthy lifestyle practices. The consumption of adaptogenic herbs periodically could enhance the body's capacity to maintain balance amid a variety of stressors [4].

*Ocimum tenuiflorum* L., also known as *Ocimum sanctum* L., is an aromatic plant (Family: Lamiaceae), widely distributed and cultivated throughout the Southeast Asian tropics [5, 6]. *O. tenuiflorum* L. is regarded within Ayurveda as the preeminent herb and is known as “The Queen of Herbs” or “Elixir of Life” due to its medicinal and spiritual properties [7]. Different parts of *O. tenuiflorum* L., specifically leaves, stem, flowers, roots, seeds, and even whole plants, are used in traditional systems of medicine like Ayurveda for the treatment of various diseases such as bronchitis, bronchial asthma, malaria, diarrhea, dysentery, skin diseases, arthritis, eye diseases, chronic fever, anxiety, cough, indigestion, hiccups, vomiting, gastric, cardiac, and genitourinary disorders, back pain, ringworm, and snake, scorpion, and insect bites [7–12].

Several studies conducted in vitro and in vivo and human experiments proved that *O. tenuiflorum* L. possesses antifertility, anticancer, antidiabetic, antifungal, hepatoprotective, cardioprotective, antiemetic, antispasmodic, analgesic, adaptogenic, antimicrobial (including antibacterial, antiviral, antifungal, antiprotozoal, antimalarial, and anthelmintic), mosquito repellent, antioxidant, anticataract, antiinflammatory, chemopreventive, radioprotective, neuroprotective, antihypercholesterolemia, antihypertensive, anticarcinogenic, analgesic, antipyretic, antiallergic, immunomodulatory, central nervous system depressant, memory enhancement, antiasthmatic, antitussive, diaphoretic, antithyroid, antiulcer, antispasmodic, antiarthritic, antistress, antileukodermal, anticoagulant, and diaphoretic properties [7–15]. These pharmacological studies have established a scientific basis for the therapeutic uses of this plant [8].

The consumption of *O. tenuiflorum* L. on a regular basis is said to prevent disease, promote general health, well-being, and longevity, and help the body and mind cope with a wide range of chemical, physical, infectious, and emotional stresses and restore physiological and psychological function [4, 8–11]. *O. tenuiflorum* L. can also provide luster to the complexion and sweetness to the voice and foster beauty, intelligence, stamina, and a calm emotional disposition [8–11]. *O. tenuiflorum* L. has long been consumed as tea herbs/a cuisine material in Vietnam and other Southeast Asian countries [5]. The best known active compounds that have been identified and extracted from *O. tenuiflorum* L. are eugenol, the major component of essential oil, and ursolic acid [9]. It has been reported that the leaves of this plant contain essential oils composed of limonene, borneol, copaene, caryophyllene, and elemol, flavonoids (apigenin, luteolin, apigenin-7-O-glucuronide, orientin, and olludistin), triterpenoids (oleanolic and ursolic acid), polyphenols, and tannins [5, 14].

Silver nanoparticles using the *O. tenuiflorum* L. leaf extract were synthesized which showed significant antibacterial activity against *E. coli* and *Staphylococcus aureus* [15–19]. The 50% ethanol extract of *O. tenuiflorum* L. leaves seemed to be nontoxic as was seen after its acute and subacute oral administrations of 200, 600, and 2000 mg/kg [14].

In the context of Nepal, two varieties of *O. tenuiflorum* L. are available. One of the varieties commonly known as Krishna Tulsi consists of purple-colored leaves and another variety known as Sri Tulsi comprises green-colored leaves. The antioxidative activity of *O. tenuiflorum* L. has been already proved, but the activity of the two varieties has not been compared. Though *O. tenuiflorum* L. is widely used traditionally to treat various disorders and its clinical efficacy has been proven, it has not been available in appropriate dosage forms.

The effervescent granules proved to be a promising vehicle for plant extracts due to their simplified processing, easy administration, rapid disintegration, good flow properties, and low cost [20]. By considering the therapeutic potentials of *O. tenuiflorum* L. and the advantages of effervescent formulation, the study aimed to evaluate the antioxidant activity of leaves of green-leafed and purple-leafed varieties of *O. tenuiflorum* L. and develop the effervescent granules of the potent extract and evaluate the quality parameters of the formulated granules.

## 2. Materials and Methods

### 2.1. Plant Material

**Figure 1:**
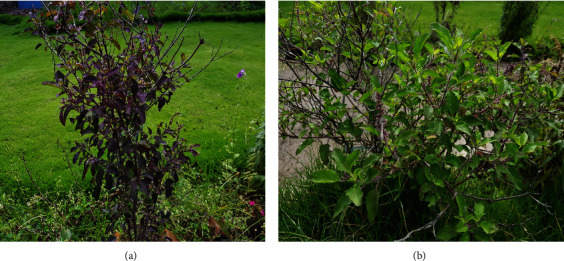
Aerial part of *O. tenuiflorum* L.: (a) purple-leafed and (b) green-leafed.

Two varieties of *O. tenuiflorum* L. as shown in Figure 1 were collected from the Kaski district of Nepal. The identification of plants was done by comparing the morphological characteristics of plant materials with the literature and confirmed by the botanist Prof. Dr. Radhe Shyam Kayastha. The authentic samples of plant materials have been preserved in the Pharmacognosy Laboratory of School of Health and Allied Sciences, Pokhara University, with crude drug voucher numbers PUCD-2017-37 and PUCD-2017-38, respectively.

### 2.2. Chemicals

All the chemicals used were of analytical grade. Ascorbic acid (HiMedia Laboratories Pvt., Ltd.), citric acid (Qualigens Fine Chemicals Pvt., Ltd.), 2,2-diphenyl-1-picrylhydrazyl (Wako Pure Chemical), ethanol (CS, India), sodium bicarbonate (Merck Pvt., Ltd.), and tartaric acid (Qualigens Fine Chemicals Pvt., Ltd.) were used for the study.

### 2.3. Equipment

A rotary evaporator (Buchi, Germany), UV spectrophotometer (UV-1800, Shimadzu, Japan), dissolution apparatus (Electrolab), and sonicator (PCi Analytics Pvt., Ltd.) were the major equipment used for the study.

### 2.4. Extraction

The plant samples were extracted using the maceration method. The ground powder (100 g) of each plant material was extracted with ethanol (2 × 700 mL) at room temperature for 24 hours. The extracts were then concentrated to dryness under a rotary evaporator. The extraction yield value of the plant extracts was calculated using the following formula:

(1) $$\begin{array}{c} \%\;yield=\frac{weight\;of\;extract\;obtained}{weight\;of\;crude\;drug\;taken}\times 100\text{.} \end{array}$$

### 2.5. DPPH Radical Scavenging Assay

DPPH radical scavenging activity was measured according to the method described by Baral and Basnet [21] with slight modifications. Two mL of ethanolic solution of each extract at various concentrations (1 *µ*g/mL, 10 *µ*g/mL, and 100 *µ*g/mL) was mixed with 2 mL of ethanolic solution of DPPH (approx. 60 *µ*M) and left for 30 minutes at room temperature. The antioxidative activity of extracts corresponding to the scavenging of DPPH radical was measured at 517 nm by the absorbance of UV spectrophotometer.

(2) $$\begin{array}{c} Radical\;scavenging\;activity\;\left(\%\right)=\frac{control\;absorbance-extract\;absorbance}{control\;absorbance}\times 100\text{,} \end{array}$$

where control was the test solution without extract. Ascorbic acid was used as the positive control.

### 2.6. Formulation of Effervescent Granules

Effervescent granules of the ethanolic extract of the purple-leafed* O. tenuiflorum* L. were formulated by the wet granulation method described by Xiang-Zhou and Sheng [22] and Forman et al. [23] with some modifications. According to Forman et al. [23], the best effervescent vehicle ratio of citric acid, tartaric acid, and sodium bicarbonate is 1 : 2 : 3.44. So, to prepare the formulation, 12 g citric acid, 24 g tartaric acid, 41.28 g sodium bicarbonate, and 2.5 g *O. tenuiflorum* L. extract were triturated into a fine powder, and then a sufficient quantity of ethanol was added to make a damp mass and passed through sieve no. 10 to obtain granules, and finally, they were dried in a hot air oven at 40°C and packed in an airtight container.

### 2.7. Evaluation of Formulated Herbal Effervescent Granules

Angle of repose, bulk density, tapped density, Carr's Index, and Hausner ratio were calculated according to Aulton and Kelvin [24], and effervescent cessation time of the formulated effervescent granules was calculated using the method described by British Pharmacopoeia [25], and stability studies were performed according to the procedure explained by Shet et al. [26].

#### 2.7.1. Angle of Repose

The angle of repose of the formulated granules was measured using a fixed funnel method. A funnel was fitted into a stand with its tip at 2.5 cm above a graph paper that is placed on a flat horizontal surface. The effervescent granules were carefully poured through the funnel until the apex of the conical pile touched the tip of the funnel. Then, the radius of the base of the conical pile was measured. The angle of repose was finally calculated using the following formula:

(3) $$\begin{array}{c} \text{Tan}\theta =\frac{h}{r}\text{,} \end{array}$$

where *θ* = angle of repose, *h* = height of the pile cone, and *r* = radius of the cone base of the pile.

#### 2.7.2. Bulk Density

Formulated granules of 15 g were introduced into a dry 100 mL cylinder, without compacting. The level of the granules was carefully marked without compacting and the unsettled apparent volume, *V*o, was read. Then, the bulk density was calculated using the following formula:

(4) $$\begin{array}{c} \rho b=\frac{M}{V0}\text{,} \end{array}$$

where *ρ*b = apparent bulk density, *M* = weight of the sample, and *V* = apparent volume of powder.

#### 2.7.3. Tapped Density

After carrying out the procedure as given in the measurement of bulk density, the cylinder containing the sample was tapped 500 times initially, followed by additional taps of 750 times until the difference between succeeding measurements was less than 2% and then tapped volume, Vf, was measured to the nearest graduated unit. The tapped density was calculated, in gram per mL, using the following formula:

(5) $$\begin{array}{c} \rho tap=\frac{M}{Vf}\text{,} \end{array}$$

where *ρ*tap = tapped density, *M* = weight of sample, and Vf = tapped volume of powder.

#### 2.7.4. Carr's Index (%)

Carr's Index (compressibility index) is a measure of the propensity of a powder to be compressed. It was determined from the bulk and tapped densities using the following formula:

(6) $$\begin{array}{c} Carr's\;Index=\frac{\left(\rho tap-\rho b\right)}{\left(\rho tap\right)}\times 100\text{,} \end{array}$$

where *ρ*tap = tapped density and *ρ*b = bulk density.

#### 2.7.5. Hausner Ratio

Hausner ratio is considered as the indirect index of ease of powder flow. It was calculated using the following formula:

(7) $$\begin{array}{c} Hausner's\;ratio=\left(\frac{\rho tap}{\rho b}\right)\text{,} \end{array}$$

where *ρ*tap = tapped density and *ρ*b = bulk density.

#### 2.7.6. Effervescent Cessation Time

A volume of 200 mL of distilled water was taken in a beaker, one dose of effervescent granules (3 g) was poured into the beaker, and effervescent cessation time and effervescent production were observed.

#### 2.7.7. Stability Studies

For stability study of the formulation, effervescent cessation time was carried out after the 1^st^ month, 2^nd^ month, and 3^rd^ month of formulation at normal room temperature, i.e., at 27 ± 2°, and accelerated temperature, i.e., at 40 ± 2°, and also the granules were examined visually for any changes in color, odor, and texture.

## 3. Results

### 3.1. Extraction of Yield Value

The percentage yield value of the ethanolic extracts of plant samples is shown in Table 1. The extract yield percentage of the leaves of purple-leafed* O. tenuiflorum* L. was found to be relatively higher than that of green-leafed* O. tenuiflorum * L.

### 3.2. DPPH Radical Scavenging Activity

**Figure 2:**
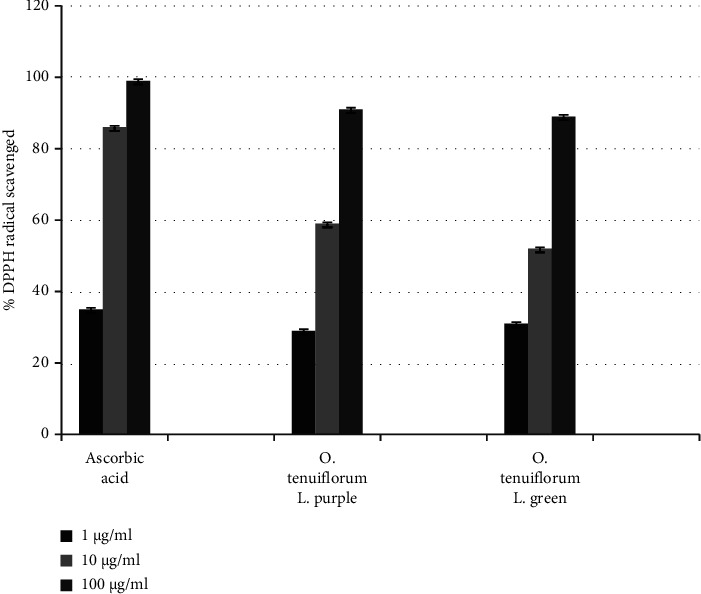
Graphical representation of percentage of DPPH radicals scavenged by ascorbic acid and *O. tenuiflorum* L. leaf extracts at 517 nm. The error bar in the diagram represents the standard deviation of three independent measurements performed in triplicate of sample size (*n* = 6).

**Figure 3:**
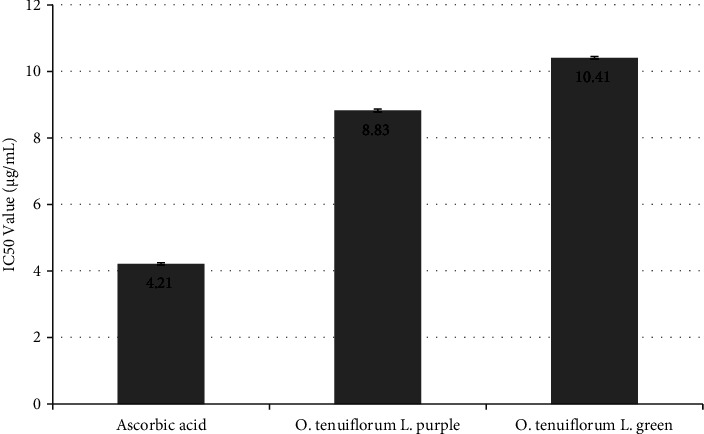
Graphical representation of IC_50_ values of ascorbic acid and *O. tenuiflorum* L. leaf extracts. The error bar in the diagram represents the standard deviation of three independent determinations performed in triplicate of sample size (*n* = 6).

The percentage of DPPH radicals scavenged by ascorbic acid and *O. tenuiflorum* L. leaf extracts at 517 nm is shown in Figure 2. The standard drug, ascorbic acid, was able to scavenge 99% of the DPPH radical at 100 *µ*g/mL concentration, whereas the ethanolic extracts of purple-leafed* O. tenuiflorum* L. and green-leafed *O. tenuiflorum* L. were able to scavenge 91% and 89% of the DPPH radicals, respectively. Similarly, Figure 3 shows IC_50_ values of ascorbic acid and *O. tenuiflorum* L. leaf extracts.

### 3.3. Formulation of Herbal Effervescent Granules

**Figure 4:**
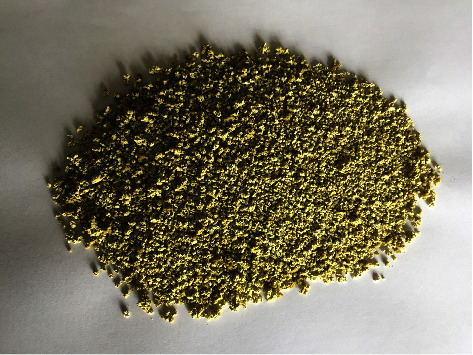
Formulated effervescent granules of the purple-leafed *O. tenuiflorum* L. extract.

The antioxidative activity of the ethanolic extract of purple-leafed* O. tenuiflorum* L. was found to be higher than that of green-leafed* O. tenuiflorum* L. Therefore, the ethanolic extract of purple-leafed* O. tenuiflorum* L. was selected to prepare the effervescent granules. The formulated effervescent granule is shown in Figure 4.

### 3.4. Evaluation of Formulated Herbal Effervescent Granules

#### 3.4.1. Angle of Repose, Bulk Density, Tapped Density, Carr's Index, Hausner's Ratio, and Effervescent Cessation Time

The result obtained from the evaluation of the physical characteristics of the formulated effervescent granules is shown in Table 2. From the study, the angle of repose, bulk density, tapped density, Carr's Index, Hausner ratio, and effervescent cessation time of the formulated effervescent granules were found to be 32.28°, 0.50, 0.53, 5.63, 1.06, and 50 sec, respectively.

#### 3.4.2. Stability Studies

The results of the stability studies of the formulated effervescent granules of the *O. tenuiflorum* L. leaf extract at normal room temperature, i.e., 27 ± 2°C, and accelerated temperature, i.e., 40 ± 2°C, for a period of three months are shown in Tables 3 and 4, respectively. The effervescent cessation time remained almost similar both at normal room temperature and at 40 ± 2°C even after 3 months. The visual characteristics (color, odor, and texture) were also found uniform as before (color: light green; odor: aromatic smell of *O. tenuiflorum* L.; texture: dry, freely flowable).

## 4. Discussion

Plants have been found as an important source of drugs. A large number of the world's populations, especially in developing countries, depend upon medicinal plants as an alternative and complementary drug therapy for various ailments [14, 27, 28]. According to the WHO, more than 60% of the world population still relies on herbals and herbal-originated medicaments for treating short and chronic ailments, and due to their widespread usages, many domestic and multinational pharmaceutical companies are inducted into the production of various herbal formulations [29]. The acceptability of herbal formulations by the public is increasing nowadays due to their affordability, effectiveness, and less toxicity. The effectiveness and therapeutic efficacy are major concerns for any type of formulation. Particularly, in addition to the above concerns, built-in quality and product standards are also key concerns for herbal formulations [30–32].

*O. tenuiflorum* L. is widely used in a number of traditional medicines. It has been found to be very effective, safe, and inexpensive in relation to its availability. Due to its proven medicinal values, *O. tenuiflorum* L. has been considered as an important plant among other herbs [33]. Therefore, *O. tenuiflorum* L. was selected for the present study to evaluate its antioxidant activity. In recent years, the search of antioxidant is of prime concern because of their use in therapy for cardiovascular diseases, ageing, cancer, arthritis, and various other diseases arising due to oxidative stress and cellular damage [34, 35].

The antioxidant activities of the prepared plant extracts were investigated by the DPPH radical scavenging assay, a very simple and reliable method to evaluate the antioxidant activity of plant extracts. DPPH forms a stable molecule when it accepts an electron or a hydrogen atom and thus can be used in the determination of radical scavenging activity of natural products [36, 37]. Free radicals, which are present in our biological systems, may oxidize all the biological molecules such as proteins, nucleic acid, and lipids and initiate degenerative diseases [38, 39]. Antioxidants can scavenge the free radicals generated within the body and also play a great role in inhibiting the chain reaction of lipid peroxidation [20, 36]. In the present study, the antioxidative properties of both standard drug and plant extracts increased with increasing concentration.

Values for the angle of repose ≤30° usually indicate a free flowing material and angles ≥40° suggest a poorly flowing material, 25–30 show excellent flow properties, 31–35 show good flow properties, 36–40 show fair flow properties, and 41–45 show passable flow properties [24]. In this study, the formulated granules had an angle of repose 32.28°, so it can be concluded that the formulated effervescent granules have good flow properties. Lower Hausner ratio (<1.25) indicates better flow properties than higher ones, between 1.25 and 1.5 showing moderate flow properties and more than 1.5 poor flow [24]. The prepared formulation had Hausner ratio of 1.05, so it also indicates that the granules have good flow properties.

Pandey et al. [40] reported that the angle of repose of formulated effervescent granules of *Martynia annua* was 33.02, and bulk density (*ρ*b) and tapped density (*ρ*tap) were 0.55 and 0.71, respectively; the compressibility index (Carr's Index) was 22 and the Hausner ratio was 1.29, which showed its moderate flow property, and the effervescent cessation time was 2-3 min, whereas in this study, all the values were found to be less than the values in the previous study and showed better flow properties and effervescent cessation time.

From stability studies, it was found that the formulated granules had similar effervescent cessation time and visual characteristics compared to initial parameters at both normal room temperature and accelerated temperature, i.e., at 40 ± 2°. This means that the formulated granules were found stable even after 3 months at both the conditions. The stability of the solid dosage form is higher compared to its liquid preparations as the potential degradation of active ingredients in aqueous media, microbial contamination, and the associated disruption of its shelf life are prevented. The effervescent granule should be dispersed in water to prepare a solution for its administration, which will provide an improvement in taste, solubility, and bioavailability of the herbal extract [41].

## 5. Conclusion

Two varieties of *O. tenuiflorum* L., green-leafed and purple-leafed, were selected to evaluate the antioxidant activity. The antioxidant activity of purple-leafed* O. tenuiflorum* L. was found higher than that of green-leafed* O. tenuiflorum* L. The formulated granules with the purple-leafed ethanolic extract showed good flow properties as depicted from the angle of repose, bulk density, tapped density, Hausner ratio, and compressibility index. The effervescent cessation time was found within the limit and the formulated granules also passed the stability test. Thus, the effervescent granules can be a promising vehicle for plant extracts due to their simplified processing, easy administration, rapid disintegration, and good flow properties.

## Figures and Tables

**Table 1:** Percentage yield value of the ethanolic extracts of *O. tenuiflorum* L. samples.

Plants	Parts used	% yield value
*O. tenuiflorum* L. (purple-leafed)	Leaves	13.48
*O. tenuiflorum* L. (green-leafed)	Leaves	10.83

**Table 2:** Physical characteristics of the formulated effervescent granules.

Characteristics	Values
Angle of repose	32.28°
Bulk density	0.50
Tapped density	0.53
Carr's Index	5.63
Hausner ratio	1.06
Effervescent cessation time	50 sec

**Table 3:** Stability studies of effervescent granules of the *O. tenuiflorum* L. leaf extract at 27 ± 2°C.

Parameters	1 month	2 months	3 months
Effervescent time (sec)	50	49.9	49.9
Visual characteristics (color, odor, and texture)	Uniform	Uniform	Uniform

**Table 4:** Stability studies of effervescent granules of the *O. tenuiflorum* L. leaf extract at 40 ± 2°C.

Parameters	1 month	2 months	3 months
Effervescent time (sec)	50	50.1	50.1
Visual characteristics (color, odor, and texture)	Uniform	Uniform	Uniform

## Data Availability

The data used to support the finding of this study are available upon request from the author.
